# The physiology of regulated BDNF release

**DOI:** 10.1007/s00441-020-03253-2

**Published:** 2020-09-18

**Authors:** Tanja Brigadski, Volkmar Leßmann

**Affiliations:** 1 Department of Informatics and Microsystem Technology, University of Applied Sciences Kaiserslautern, D-66482 Zweibrücken, Germany; 2 Institute of Physiology, Otto-von-Guericke University, D-39120 Magdeburg, Germany; 3 Center for Behavioral Brain Sciences, Magdeburg, Germany

**Keywords:** BDNF release, Neurotrophins, Secretion

## Abstract

The neurotrophic factor BDNF is an important regulator for the development of brain circuits, for synaptic and neuronal network plasticity, as well as for neuroregeneration and neuroprotection. Up- and downregulations of BDNF levels in human blood and tissue are associated with, e.g., neurodegenerative, neurological, or even cardiovascular diseases. The changes in BDNF concentration are caused by altered dynamics in BDNF expression and release. To understand the relevance of major variations of BDNF levels, detailed knowledge regarding physiological and pathophysiological stimuli affecting intra- and extracellular BDNF concentration is important. Most work addressing the molecular and cellular regulation of BDNF expression and release have been performed in neuronal preparations. Therefore, this review will summarize the stimuli inducing release of BDNF, as well as molecular mechanisms regulating the efficacy of BDNF release, with a focus on cells originating from the brain. Further, we will discuss the current knowledge about the distinct stimuli eliciting regulated release of BDNF under physiological conditions.

## Introduction

### BDNF in health and disease

The neurotrophic factor BDNF plays an important role for the development of brain circuits, the formation and maintenance of neuronal morphology, brain architecture and for synaptic, as well as neuronal network plasticity (Edelmann et al. 2014; Gottmann et al. 2009; Huang and Reichardt 2001; Klein 1994; Lessmann and Brigadski 2009; Park and Poo 2013). Consequently, BDNF crucially regulates learning and memory processes in young and adult mammals (see, e.g., Boschen and Klintsova 2017; Gomez-Pinilla and Vaynman 2005) such that imbalances in BDNF levels and downstream signaling via its cognate TrkB tyrosine kinase receptor are associated with neurodegenerative and psychiatric diseases, like Alzheimer’s disease, major depressive disorder (Castren and Hen 2013), or schizophrenia (Mohammadi et al. 2018). Moreover, BDNF signaling also contributes to physiological functions of the heart and the vasculature and is involved in disorders like coronary artery disease (Ejiri et al. 2005; Jin et al. 2018; Kaess et al. 2015), diabetes mellitus (Eyileten et al. 2017; Suwa et al. 2006), inflammatory diseases such as asthma (Prakash and Martin 2014), different types of cancer (Chopin et al. 2016; Radin and Patel 2017), as well as pain sensation (Deitos et al. 2015; Haas et al. 2010; Laske et al. 2007; Merighi et al. 2008; Sapio et al. 2019). Furthermore, BDNF levels in preterm neonates differ from BDNF levels in full-term neonates (Malamitsi-Puchner et al. 2004), thereby affecting cognitive development in early postnatal life (Chau et al. 2017) and potentially being associated with children’s mental diseases such as autism spectrum disorders (Qin et al. 2016; Zheng et al. 2016).

### Measurements of BDNF as a diagnostic marker

In human medical studies, ELISA or Western blot-based measurements of BDNF protein levels in body fluids (e.g., blood or liquor) or tissue samples are considered as a potential proxy of intact brain (or other organ) function and associated diseases. As a result, great efforts are being made to utilize measurements of the BDNF levels as a potential marker for health status, diagnosis and prognosis of diseases and therapeutic outcome (Balietti et al. 2018). Variations in BDNF genotype (compare Intracellular sorting of BDNF in neurons section) are considered to be covariate information in such approaches (Shen et al. 2018). In the blood, the secretory protein BDNF is mainly stored in platelets (Fujimura et al. 2002). However, little is known about the original source of circulating BDNF in blood plasma (Dawood et al. 2007; Klein et al. 2011; Krabbe et al. 2007; Rasmussen et al. 2009; Serra-Millas 2016). BDNF is prominently expressed in the brain, in hippocampus, cortex, amygdala and striatum but also in hypothalamus (reviewed in Edelmann et al. 2014). It is also expressed and released from endothelial cells (Cefis et al. 2020), cells of the immune system (lymphocytes, microglia), megakaryocytes (Chacon-Fernandez et al. 2016; Tamura et al. 2012) and others, like smooth muscle cells (reviewed in Edelmann et al. 2014). Activity-dependent release of BDNF from hypothalamic neurons, which are not shielded from the blood stream by the blood-brain barrier, likely contributes to blood BDNF levels. Transport of BDNF across the blood brain barrier was, although controversially discussed, also described for neurotrophins like BDNF (Pan et al. 1998; Rasmussen et al. 2009; Seifert et al. 2010). Therefore, BDNF released from neurons as a consequence of neural network activity in the brain can act locally by tuning the strength of the neural network but it may also act globally by affecting the function of other organs distant from the brain after diffusion into the bloodstream. Vascular endothelial cells as well as cells of the immune system represent another natural source of BDNF, which likely contribute to the regulation of BDNF levels in the blood (Kerschensteiner et al. 1999; Marie et al. 2018; Nakahashi et al. 2000). However, stimuli-inducing release of BDNF from non-neuronal tissue like platelets and vascular endothelial cells are not well understood.

In the human brain, analysis of post mortem tissue is currently almost the only way to estimate changes in BDNF levels. Decreased levels of BDNF have been detected post mortem in the cortex, hippocampus and nucleus basalis of Meynert in patients suffering from Alzheimer’s disease (Holsinger et al. 2000; Tapia-Arancibia et al. 2008), while region-specific down but also upregulation of BDNF has been shown in major depressive disorder patients (Krishnan et al. 2007; Pandey et al. 2008). However, to understand the relevance of change in BDNF levels in the brain, non-neuronal tissue and blood, a more detailed knowledge of the distinct stimuli-regulating BDNF expression and release in the different cell types is important. Most studies providing information about the molecular and cellular mechanism regulating intra- and extracellular BDNF levels have thus far been performed in cells originating from the brain. The role of distinct subcellular BDNF release sites as well as release kinetics, e.g., for cell survival or synaptic plasticity is best studied in neurons (reviewed, e.g., in Edelmann et al. 2014). Therefore, this review will discuss recent advancements in cell-type specific activity-dependent release of BDNF, with a focus on BDNF release from cells originating from the brain. We will summarize the stimuli inducing or amplifying the release of BDNF as well as the molecular regulation of BDNF secretion and discuss the role of BDNF secretion in the context of synaptic plasticity and specific diseases.

## Synthesis, processing and sorting of BDNF

### General aspects of BDNF synthesis

The secretory protein BDNF is expressed in neuronal and non-neuronal cells (Katoh-Semba et al. 1997; Maisonpierre et al. 1990; Phillips et al. 1990). In neuronal cells, BDNF-immunoreactivity was found in several regions of the central nervous system as well as in the peripheral and enteric nervous system (Barakat-Walter 1996; Conner et al. 1997; Ernfors et al. 1990; Hoehner et al. 1996; Wetmore and Olson 1995; Yan et al. 1997; Zhang et al. 2007; reviewed, e.g., in Lessmann et al. 2003; Lewin and Barde 1996). In non-neuronal tissue, BDNF is synthesized in cells of the immune system, like T cells, B cell and monocytes (Kerschensteiner et al. 1999; Nakahashi et al. 2000), muscle cells (Matthews et al. 2009; Mousavi and Jasmin 2006), the heart (Donovan et al. 2000; Maisonpierre et al. 1990), thymus, liver and spleen (Katoh-Semba et al. 1997). The tissue-specific expression of BDNF is developmentally regulated (Ernfors et al. 1990; Katoh-Semba et al. 1997). Furthermore, physiological as well as pathophysiological conditions and interventions such as exercise, hypoxia, stress, epileptic seizures and ischemia increase expression of BDNF in a tissue-specific manner (Giannopoulou et al. 2018; Lippi et al. 2020; Thomas et al. 2016; Wetmore et al. 1994).

In humans, the BDNF gene is located on chromosome 11p14.1 consisting of 11 exons (I–XI) and nine promotors that regulate the developmental and regional expression of multiple alternatively spliced mRNA isoforms (Pruunsild et al. 2007; Timmusk et al. 1993). Only exon IX at the 3′ end of the gene locus contains the major coding sequence for the BDNF precursor protein (Pruunsild et al. 2007). This BDNF coding sequence is translated into a pre-pro-protein, with an N-terminal pre-domain that guides the mRNA to the rough endoplasmic reticulum (rER). The pre-sequence is cleaved of co-translationally and the immature uncleaved pro-BDNF protein is synthetized into the ER (compare Lessmann and Brigadski 2009). Importantly, transcription of the BDNF gene into a set of distinctly spliced mRNA variants (Aid et al. 2007; Pruunsild et al. 2007) is tightly regulated by electrical activity-induced Ca^2+^ elevation in neurons (e.g., (Castrén et al. 1998). The distinct mRNA variants can be transported into dendrites (Baj et al. 2013), where local translation and release of BDNF can be confined to especially active dendritic stretches thereby promoting synaptic plasticity in a BDNF-dependent fashion. This makes BDNF ideally suited to shape developing and fine tune mature synaptic circuits through activity-dependent local synthesis and secretion (compare chapter “Physiological context of regulated release of BDNF section” in this review, see also Johnestone and Mobley 2020).

### Posttranslational processing of BDNF

Secretory proteins are posttranslationally processed by different enzymes such as endo- or exopeptidases or glycosyltransferases (reviewed in Thomas 2002). The incidence of the diverse posttranslational modifications depends on the entire set of modifying proteins expressed by a specific cell, as well as on the intracellular ionic composition in cytoplasm, ER and Golgi (Creemers 2002; Gidalevitz et al. 2013). Characteristic features of secreted proteins, such as biological activity, half-life time, or affinity to specific intracellular binding partners are modified by these events. The best-known example for a precursor protein (pro-protein) that undergoes multiple posttranslational modification steps is the hormone pro-opiomelanocortin (POMC). POMC is the precursor of a variety of hormones like ACTH, MSH, or beta-endorphin. These diverse peptides are generated after endoproteolytic cleavage of the precursor protein POMC by subtilisin-like prohormone convertases PC1 or PC2. The thereby generated new N- and C-termini are further processed by exopeptidases, acetyltransferases and/or peptidyl-glycine alpha-amidating monooxygenases (compare Lessmann and Brigadski 2009). However, the corresponding processing of BDNF has not been intensively investigated so far. It is known that the 32-kDa precursor proBDNF can be processed endoproteolytically within the pro-domain by the enzyme subtilisin/kexin-isozyme 1 (SKI-1), generating a 28 kDa protein (Seidah et al. 1999). In addition to the 28-kDa cleavage product of the BDNF precursor, the 14-kDa mature BDNF is generated after cleavage of proBDNF by furin-like protein convertases PACE4, PC1, or PC5 (Mowla et al. 2001; Seidah et al. 1996). Inhibition of furin-like enzymes prevents the synthesis of the 14 kDa mature BDNF without any effect on the generation of the 28 kDa cleavage product (Mowla et al. 2001). Furthermore, extracellular processing of proBDNF to the 14 kDa mature BDNF isoform by the tissue-type plasminogen activator (tPA)/plasmin proteolytic system and by matrix metalloproteinases (MMP) 3, 7 and 9 has been described (Gray and Ellis 2008; Lee et al. 2001; Pang et al. 2004). Further modifications like N-glycosylation and glycosulfation within the prodomain of BDNF increase the half-life time of the protein (Mowla et al. 2001). These posttranslational modifications may also be a prerequisite for interactions of BDNF with chaperones or sorting receptors that guide the secretory protein BDNF predominantly to vesicles of the regulated secretory pathway (reviewed in Leßmann and Brigadski 2009).

### Intracellular sorting of BDNF in neurons

Up to now, two sorting proteins have been identified for BDNF. Both, the chaperone sortilin and the exoproteolytic enzyme carboxypeptidase E, bind to the BDNF pro-domain and play an important role in sorting of BDNF to vesicles of the regulated pathway of secretion (Chen et al. 2005; Lou et al. 2005; Kojima et al. 2020). Interestingly, transfection of neurons with a cDNA construct, in which the pre-pro-domain of BDNF is fused to the mature part of the neurotrophin-4 (NT-4) redirects this BDNF-NT-4 chimera efficiently into vesicles of the regulated secretory pathway, whereas wildtype NT-4 is predominantly located in vesicles of the constitutive secretory pathway (Brigadski et al. 2005). This indicates the importance of the BDNF pro-domain for sorting of the protein to the regulated secretory pathway. The significance of the pro-domain for correct sorting of BDNF to secretory granules is also stressed by the commonly observed Val66Met single-nucleotide polymorphism (SNP) located in the BDNF pro-domain. This SNP (carriers in Europe: ~ 60% Val/Val (WT); ~ 35% Val/Met; ~ 5% Met/Met) is associated with reduced sorting of Met-BDNF to vesicles of the regulated secretory pathway, resulting in impaired activity-dependent secretion of BDNF from neuronal cells (Egan et al. 2003). However, this SNP-dependent reduced sorting to secretory granules does not seem to affect all BDNF transcripts to the same extent (Jiang et al. 2009). In addition to the major sorting of BDNF to the regulated secretory pathway (Brigadski et al. 2005), BDNF is also directed to vesicles of the constitutive secretory pathway. However, little is known about the molecular composition of constitutively released vesicles as well as the function of BDNF released in a constitutively manner.

## Physiological context of regulated release of BDNF

Just as there exist different cell types expressing and releasing BDNF, diverse stimuli are known to induce regulated release of BDNF. The best characterized stimuli are patterns of neuronal electrical activity like prolonged depolarization, high-frequency stimulation (HFS), or theta-burst stimulation (TBS) that trigger BDNF release in developing and mature neurons (Edelmann et al. 2014) (compare Stimuli-triggering BDNF release from neurons section) (Table 1). Release stimuli in electrically non-excitable cells differ, of course, from the major release stimuli in neuronal cells. Thus, binding of extracellular nucleotides (Coull et al. 2005; Trang et al. 2009; Ulmann et al. 2008; Vignoli and Canossa 2017), pro-inflammatory factors (Jornot et al. 2007), or neuropeptides (Lopez-Benito et al. 2018) to their corresponding receptors has been shown to induce release of BDNF from astrocytes and microglia (compare BDNF release from astrocytes section and BDNF release from microglia section) (Table 2). Next to the different physiological BDNF-releasing stimuli, knowing the subcellular sites where BDNF secretion can take place is important to understand cell-type and release-site specific functions of BDNF, e.g., during neuronal development, synaptic plasticity processes, or in case of disease (e.g., Hartmann et al. 2001; Matsuda et al. 2009; Xia et al. 2009; reviewed in Edelmann et al. 2014; Leßmann and Brigadski 2009; Park and Poo 2013).

**Table 1:** Somato-dendritic release of BDNF

**Site**	**Ref.**	**Species / type of preparation**	**Method**	**Release stimulus**	**Pharmacology / molecular mechanism / time course of BDNF release**
**Somato-dendritic release of BDNF**					
Wilson Horch et al. 1999		Organotypic slices	Postsynaptic BDNF-myc; TrkB-IgG		postsynaptic BDNF overexpression alters density and stability of spines
Hartmann et al. 2001		Rat; hippocampal cultures	Live cell imaging, BDNF-GFP	Elevated extracellular K^+^	Reduced by: TTX; 0mM Ca^2+^; 2 mM Cd^2+^/2 mM Ni^2+^; Not affecetd: APV, DNQX, LY 341495 (mGluR antagonist)
				HFS	Reduced by: APV, DNQX
Kojima et al. 2001		Rat; hippocampal cultures	Live cell imaging BDNF-GFP	Elevated extracellular K^+^	Reduced by: TTX
Brigadski et al. 2005		Rat; hippocampal cultures	Live cell imaging BDNF-GFP	Elevated extracellular K^+^	Dependency of release kinetics on intragranular pH Comparison of neurotrophin and neurotransmitter release kinetics
Arancibia et al. 2007		Adult rat; push pull perfusion + ELISA	ELISA	Intra supraoptic nucleus osmotic stimulation (1M NaCl for 10 min)	*In vivo* measurement of endogenous BDNF secretion
Kolarow et al. 2007		Rat; hippocampal cultures	Live cell imaging BDNF-GFP	Elevated extracellular K^+^	Kiss and run fusion events Reduced by: 0 mM Ca^2+^; nifedipine; thapsigargine; CPA; ryanodine; KN-93; KN-62; Rp-cAMP Not affected by: TTX; KN-92; 8-Br-cAMP; K252a
Kuczewski et al. 2008		Rat; hippocampal cultures	Live cell imaging BDNF-GFP	Depolarizations	Reduced by: GDPβS; Cd^2+^ Not affected by: QX314 (Na^+^ channel blocker)
				bAP (8 b-APs at 5 Hz)	Reduced by: QX314 (Na^+^ channel blocker); Cd^2+^ Not affected by: thapsigargine
				4-AP (10 min - blocker of Kv1)	Reduced by: NBQX +APV + bicuculline; TTX
Dean et al. 2009		Rat: E18-20 Mice: P1-3; neuronal cultures	Live cell imaging BDNF-pHluorin	Elevated extracellular K^+^	Increased after syt-IV knockout; Reduced after syt-IV overexpression Dendritic BDNF release regulates EPSC amplitude
Fiorentino et al. 2009		Rat; hippocampal culture	Live cell imaging BDNF-GFP	Baclofen (10μM for 500sec - significant after 5 min application)	Reduced by: CGP55845 (GABA_B_-receptor antagonist); Cd^2+^; Not affected by: NBQX, APV, bicuculline
Matsuda et al. 2009		Rat E18-20, neuronal cultures	Live cell imaging BDNF-GFP	HFS field stimulation	Reduced by: CNQX, APV; CNQX; APV; nimodipine; Cd^2+^ Not affected by: bafilomycin; dynasore; K252a
				Loose patch stimulation	Reduced by: nimodipine Not affected by:CNQX + APV; CNQX; APV
				TBS	Highest efficacy of BDNF release
Wit et al. 2009		Mouse; cortical culture;	Live cell imaging BDNF-sp-pHluorin	Elevated extracellular K^+^	Transient and persistent release (deposit)
Xia et al. 2009		Rat; Hippocampal Cultures	Live cell imaging BDNF-GFP	Elevated extracellular K^+^	Kiss and run fusion events; Release probability in soma is higher than in neurites; Onset of release in soma is delayed in neurites compared to soma Blocked by verapamil (L-Type VGCC) Not affected by conotoxin (N/Q-Type); agatoxin (P-type)
Yang et al. 2009		Xenopus; neuron myocte coculture	selective knockdown of BDNF	Repetitive depolarization of myocyte	Transsynaptic action of proBDNF ➔ p75 activation ➔ synaptic retraction
Jakawich et al. 2010		Rat; hippocampal cultures	TrkB-IgG; BDNF knockdown	Inhibition of AMPAR (3h)	Transcription dependent postsynaptic BDNF release mediates presynaptic increase in mEPSC frequency
Waterhouse et al. 2012		Mouse; hippocampal cultures	Dendritic myc-IR	potassium	long 3′ UTR controls dendritic localization of BDNF mRNA
Adachi et al. 2013		Rat; cortical cultures and acute cortical slices	BDNF-IR ELISA	Basal secretion	Reduced by PCP (1μM for 6h) (NMDAR blocker) ➔ although increased somatic and dendritic accumulation of BDNF
				Glutamate (15 min)	Reduced by: TTX
Leschik et al. 2013		Embryonic stem cell derived neurons	Live cell imaging BDNF-GFP	Elevated extracellular K^+^	Similar release properties of BDNF–GFP in ESC-derived neurons and transiently transfected hippocampal neurons
Petoukhov et al. 2013		Rat; hippocampal cultures	Live cell imaging	4-AP	BDNF is localized in progranulin positive vesicles in dendrites and axons Release reduced by: Ca-free solution; CdCl_2_
Kolarow et al. 2014		Rat; hippocampal cultures	Live cell imaging	potassium	Reduced by SNP (NO-donor) Not affected by: L-NMMA (NOS-inhibitor)
Lu et al. 2014		Rat; hippocampal slices; embryonic hippocampal cultures	TrkB-IgG; live cell imaging (BDNF-GFP)	Timing: bAPs + iontophoretic glutamate pulses	No release by bAPs or iontophoretic glutamate alone Reduced by: APV
Edelmann et al. 2015		Organotypic slice; hippocampal slices	Live cell imaging	20 Hz + 8-Br-cAMP	
				Elevated K^+^	
Shimojo et al. 2015		Cortical cultures	Live cell imaging; BDNF-pHluorin	Elevated extracellular K^+^	Axonal and dendritic BDNF-containing vesicles are localized to Syb2, SNAP25 and SNAP47 Full vesicle collapse is reduced in SNAP 47 kd cultures Partial vesicle collapse is reduced in axons of SNAP 47 kd cultures
Wong et al. 2015		Neuronal culture	BDNF-quantum dots	TBS	Axonal and dendritic localization of endocytosed BDNF-QD reduced by Cd2+; CNQX; APV; CNQX + APV; syt-6 siRNA; Complexin siRNA
			BDNF-GFP	TBS	Increased after syt4-siRNA Not affected by syt6-siRNA
Baj et al. 2016		Rat; hippocampal neurons	ELISA	Potassium (3h)	Translation-dependent BDNF increase Release reduced by: cycloheximide
Eckenstaler et al. 2016		Rat; hippocampal cultures; P0-P3, DIV 11-13; BDNF-GFP	Live cell imaging BDNF-GFP	Elevated extracellular K^+^	CAPS1 siRNA: Increase of intragranular pH from 5.8 to 6.7 Reduced incidence of fusion events from 20 % to 10 % Decreased amount of released BDNF per vesicle Not affected: cytosolic pH
					Inhibition of V-ATPase: reduced incidence for fusion events and reduced BDNF content release
Harward et al. 2016		Cortical cultures; organotyipic slice	TrkB-IgG; live cell imaging; postsynaptic BDNF deletion	Glutamate uncaging	Reduced by APV; APV + NBQX; CN21 (inhibitor of CAMKII) Not affected by: NBQX
Henry et al. 2018		Rat; hippocampal cultures	BDNF knockdown	Inhibition of AMPAR (3h)	mTor dependent translation of postsynaptic BDNF release mediates presynaptic increase in mEPSC frequency
Lin et al. 2018		Hippocampal slice	Deletion of BDNF in CA1 region	100 Hz-HFS	“Postsynaptic release”: LTP maintenance + increase in presynaptic release probability
Brigadski et al. 2019		Rat; hippocampal cultures	Live cell imaging	Elevated extracellular K^+^	comparable release kinetics in response to high potassium depolarization vs. electrophysiological stimulation
				Depolarization	
				bAP	
Leschik et al. 2019		BDNF-GFP-knockin mouse, hippocampal cultures, expression regulated by endogenous BDNF promotor	Live cell imaging BDNF-GFP	Elevated extracellular K^+^	Identical release properties of endogenous BDNF–GFP and overexpressed BDNF-GFP 20 % fusion events 60 % content release Max. of fusion events within 20 s of stimulation; fusion events at 100 s still prominent
Persoon et al. 2019		Neuronal cultures	Live cell imaging	Elevated K^+^	Rab3a, RIM1/2, Munc13 localized to BDNF-containing granules
**BDNF release from axons**					
Kohara et al. 2001		Mouse, cortical cultures	GFP-IR	Analysis of somatic BDNF-GFP Endocytosis	Increased by: picrotoxin for 48 hours Reduced by: TTX (48 h); TrkB IgG (48 h)
Zakharenko et al. 2003		Hippocampal slice	Deletion of BDNF	200Hz-HFS	
Dean et al. 2009		Rat: E18-20 Mice: P1-3	Live cell imaging; BDNF-pHluorin	Potassium	Increased after syt-IV knockout; Reduced after syt-IV overexpression Regulation of mEPSC frequency
Matsuda et al. 2009		Rat, E18-20; neuronal cultures	Live cell imaging; BDNF-pHluorin	TBS	transient fusion pore opening
				50Hz (3min) or TBS (36 trains)	Induction of net BDNF release
				Field stimulation	Analysis of transient fusion events Reduced by: Bafilomycin; Dynasore; Cd^2+^ Not affected by K252a, TrkB-Fc; CNQX, APV; CNQX; APV; nimodipine
				Loose patch stimulation	Analysis of transient fusion events Not affected by: nimodipine; CNQX, APV; CNQX; APV
Shinoda et al. 2011		Mouse; hippocampal culture	Live cell imaging; BDNF-pHluorin	Potassium	Increased after: CAPS2 transfection Reduced after : CAPS2 knockout
Sadakata et al. 2012		Mouse, hippocampal granule cell cultur	GFP-IR	Potassium	Reduced after : CAPS2 with deletion of exon3
Petoukhov et al. 2013		Rat; hippocampal cultures	Live cell imaging	4-AP	BDNF is localized to progranulin positive granules in dendrites and axons Release reduced by: Ca-free solution; CdCl
Shimojo et al. 2015		Cortical culture; P1	Live cell imaging; BDNF-pHluorin	Potassium	Axonal and dendritic BDNF-containing vesicles are localized to Syb2, SNAP25 and SNAP47 Full vesicle collapse is reduced in SNAP 47 kd cultures partial vesicle collapse is reduced in axons of SNAP 47 kd cultures
Lin et al. 2018		Hippocampal slice;	Deletion of BDNF in CA3	100Hz-HFS	“presynaptic release”: LTP-induction + LTP maintenance + increase in presynaptic release probability
Park 2018		Mouse; corticostriatal slice	BDNF-pHluorin	TBS	reduced by APV (presynaptic NMDAR); CPA (depletion of internal Calcium store); requires GluN1 subunit not affected by dopamine
				HFS without Mg^2+^ in extracellular solution	reduced by APV
Persoon et al. 2019		Neuronal culture	Live cell imaging	Potassium	Rab3a, RIM1/2, Munc13 localized to BDNF-containing granules

List of references for somatic and dendritic release of BDNF. *4-AP* 4, amino-pyridine; A2AR, adenosine A2A receptor; *Aβ*, amyloid-β; *ACPD*, 1-amino-1,3 dicarboxy cyclopentane; *ATP*, adenosine triphosphate; *Br-cAMP*, brom-adenosine 3′5′-cyclic monophosphate; *CAMKII*, Ca^2+^-calmodulin-dependent protein kinase II; *CAPS*, calcium-activated protein for secretion; *CNQX*, 6-cyano-7-nitroquinoxaline-2,3-dione; *D-APV*, D(−)-2- amino-5-phosphonovalerate; *DIV*, days in vitro; *E*, embryonic day; *ELISA*, enzyme-linked immunosorbent assay; *EP2*, prostaglandin E receptor subtype2; *GABABR*, g aminobutyric acid; *HFS*, high-frequency stimulation; *ITI,* intertrain-interval; *IP3*, inositol triphosphate; *IR*, immunreactivity; *LPS*, lipopolysaccharide; *NaV*, voltage-gated sodium channel; *NBQX*, 2,3-dihydroxy-6- nitro-7-sulfamoyl-benzo(F)quinoxaline; *NMDAR*, N-methyl d-aspartate receptor; *P*, postnatal day; *P2XR*, P2X purino receptor; *p38MAPK*, p38 mitogen-activated protein kinase; *PAR1-AP*, protease-activated receptor activating peptide, *PCP*, phenylcyclidine; *PKA*, protein kinase A; *PKC*, protein Kinase C.; *PLC*, phospholipase C; *SpH*, superecliptic pHluorin; *syt-IV*, synaptotagmin-IV; *TBS*, theta-burst stimulation; *TTX*, tetrodotoxin; *VGCC*, voltage-gated calcium channels; *TRPC*, transient receptor potential channel

**Table 2:** BDNF release from astrocytes

Site	Ref.	Species/type of preparation	Method	Release stimulus	Pharmacology/molecular mechanism/time course of BDNF release
Saha et al. 2006		Rat, astrocytic cultures	ELISA	TNF-alpha	
Jean et al. 2008		Rat, basal forebrain astrocytic culture	Western blot, ELISA	Glutamate (100 μM for 10 min)	
				ACPD (10 μM for 10 min)	Reduced by: U73122 (PLC inhibitor); 2ABP (IP3 inhibitor); BAPTA-AM
Baumbauer et al. 2009		Rat	TrkB IgG	Tailshock	
Hutchinson et al. 2009		Human Astrocytoma cells	ELISA	Prostaglandin E_2_ (> 0.1 μM for 24 h)	Reduced by: H-89 (PKA inhibitor)
				butaprost (10 μM for 24 h-EP_2_ selective agonist)	Reduced by: H-89 (PKA inhibitor)
				Forskolin (10 μM for 24 h)	
Giralt et al. 2010		Mouse, astrocytic cultures	ELISA	TNF-alpha	
Hou et al. 2011		Mouse, astrocytic cultures	ELISA	Aβ42 oligomers (30 μM for 48 h)	
Gimenez-Cassina et al. 2012		SH-SY5Y neuro-blastoma cell	ELISA	Inhibition of GSK3 (24 h)	
Su et al. 2012		Rat, cortical primary astrocytes	ELISA	Progesterone (P4) (0.1 nM for 18 h)	Reduced after siRNA against progesterone receptor membrane component 1 (Pgrmc-1)
Zhang et al. 2012		Rat, primary astrocytes	ELISA	Resveratrol (100 μM, 24 h)	
Hong et al. 2016		HD mice, primary astrocytic cultures; brain slices	ELISA	Elevated K^+^	Reduced BDNF release in htt expressing cultures Reduced amount of docked BDNF-containing vesicles in htt expressing cultures –> rescued after Rab3a overexpression Increased BDNF release in Rab3a overexpressing cultures Reduced association between Rab3-GAP1 and Rab3a by mHtt
Sun et al. 2016		Rat; C6 glioma cells; SH-SY5Y neuro-blastoma cell	Western blot	Progesterone	Increases the ratio of mature to pro-BDNF released from glia ➔ Reduced after siRNA against progesterone receptor membrane component 1 (Pgrmc-1)
Sen et al. 2017		Human primary astrocytes	Western blot	ApoE3 + cholesterol (4 h)	Predominantly release of proBDNF
				ApoE2 + cholesterol (4 h)	Predominantly release of mBDNF
				Basal release	Not affected by ApoE4 + cholesterol or cholesterol
Vignoli and Canossa 2017		Cortical astrocytic cultures	ELISA	Elevated K^+^	
				Glutamate	
				ATP	
Datta et al. 2018		Astrocytic culture from forebrain, midbrain, hindbrain	ELISA	OHDA	Reduced by L-NAME (NO synthase inhibitor)
Stahlberg et al. 2018		Astrocytic cultures	BDNF-mRFP		Neuronal BDNF-mRFP is endocytosed by astrocytes via TrkB-receptor and is sorted to rab7-positive late endosomal compartment and LAMP1-positive lysosomal compartment
Su et al. 2019		Rat, primary culture of Schwann cells	ELISA	TNF (3 h)	Reduced by 5 BDBD (P2x4R antagonist); TNP-ATP (P2x1-4R antagonist) Not affected by PPADS (P2x1,2,3,5,7 antagonist)
Release from microglia					
Ref.		Species/type of preparation	Method	Release stimulus	Pharmacology/molecular mechanism/time course
Nakajima et al. 2002		Rat, primary microglial culture	Western blot	C8-ceramide (16 h) or LPS (16 h)	Reduced by bisindolylmaleimide (PKC inhibitor)
Coull et al. 2005		Rat, primary microglial cultures	ELISA	ATP (10 μM for 5 h)	Reduced by TNP-ATP (P2X receptor inhibitor)
Hutchinson et al. 2009		Human microglial cells; ELISA		Prostaglandin E_2_ (> 0.1 μM for 24 h)	Reduced by H-89 (PKA inhibitor)
				Butaprost (10 μM for 24 h-EP_2_ selective agonist)	Reduced by H-89 (PKA inhibitor)
				Forskolin (24 h)	
Trang et al. 2009		Rat, primary microglial culture	ELISA	ATP (50 μM for 5–300 min)	Reduced by: calcium-free solution; siRNA for P2X4A; TNP-ATP (P2x1-4R blocker); SB203580 (p38MAPK inhibitor) Not affected by: thapsigargine; transcriptional inhibitor; translational inhibitor
				ATP (50 μM for 60 min)	Reduced by: calcium-free solution; siRNA for P2X4A; TNP-ATP (P2x1-4R blocker); SB203580 (P38MAPK inhibitor); transcriptional inhibitor, translational inhibitor Not affected by: thapsigargine
Gomes et al. 2013		Murine N9 microglial cells	ELISA	Lipopolysaccharide (LPS, 100 ng/mL for 6 h)	Reduced by: SCH58261 (adenosine A_2A_ receptor antagonist); adenosine deaminase; H-89 (PKA antagonist); forskolin; 8-Br-cGMP
				CGS21680 (30 nM for 6H-A2AR agonist)	Reduced by: SCH58261 (adenosine A2A receptor antagonist); LPS
				Forskolin (1 μM)	Reduced by: LPS
				8 Br-cAMP (5 μM)	Reduced by: LPS
				Chelerythrine (PKC inhibitor)	Not affected by: LPS
Ferrini et al. 2013		Rat, primary microglial culture	ELISA	Morphine (100 nM every day for 5 days)	Reduced by naloxone (opioid receptor antagonist)
Zhou et al. 2019		Cultured spinal cord slice	ELISA	CSF1 (6 h)	Reduced by SB 203580 (p38 MAPK antagonist)
Long et al. 2020		BV2 microglial culture	ELISA	ATP (120 min)	Reduced by: 5 BDBD (P2x4A antagonist); SB203580 (p38 MAPK antagonist)
Zhou et al. 2020		Mouse; primary microglial cultures	ELISA	IL-4 (12 h)	Reduced by HA-TPSO (fusion construct of translocator protein (TPSO); FGIN-1-27 (TPSO agonist)

List of references for astrocytic and microglial release of BDNF (abbreviations: please see table legend of Table 1)

### Stimuli-triggering BDNF release from neurons

In the central nervous system, BDNF is important for the development of brain circuits and for synaptic as well as for network plasticity processes in the adult brain. These processes require fine-tuning of synaptic activity and structural synaptic rearrangements in an input specific manner. The spatially restricted release of BDNF is ideally suited for this purpose. Since early neuronal networks of immature neurons differ from functionally mature synaptic circuits, it is not surprising that distinct electrical activity patterns are efficient to induce release of BDNF and that the efficacy of releasing BDNF depends on the developmental stage of the neurons as well as on the specific brain area that is investigated.

#### Stimuli-triggering BDNF release from developing neurons

In developing hippocampus, a sequence of three different electrical activity patterns was described to be important to form synaptic circuitry (Crepel et al. 2007; Egorov and Draguhn 2013; Luhmann and Khazipov 2018). First, intrinsically active neurons generate brief L-type voltage-gated Ca^2+^-channel (VGCC)-mediated spikes at embryonic stage E16-E19, before an ensemble of neurons coupled by gap-junctions generates synchronous non-synaptic, long lasting calcium-plateaus, called synchronous plateau assemblies (SPA). These SPAs are replaced by giant depolarizing potentials (GDPs), which represent a spontaneous pattern of network activity via chemical synaptic interactions. GDPs consist of large depolarizations associated with a burst of action potentials. These depolarizations last several hundreds of milliseconds, propagate by synaptic interactions as waves through the developing hippocampus and occur at low-frequency (0.003–0.06 Hz) in rodents during the first week of postnatal life (Egorov and Draguhn 2013; Luhmann and Khazipov 2018). GDPs have also been described for other developing brain areas like the neocortex, thalamus, or spinal cord. Development of cortical networks is known to progress in a similar manner as in hippocampal networks (Egorov and Draguhn 2013). However, the time course of onset of cortical development is shifted to postnatal stages (Hanganu-Opatz 2010; Kilb et al. 2011). Interestingly, the onset of BDNF expression in the rodent hippocampus becomes apparent already at E15.5 and differs from the onset of BDNF expression in cortical neurons, which appears at P4 (Baquet et al. 2004; Katoh-Semba et al. 1997). Furthermore, these electrically activity patterns (intrinsically active neurons, SPAs and GDPs) depend on activation of L-type VGCCs. Consistent with this, the release of BDNF has been reported to depend on Ca^2+^-influx via L-type voltage-gated calcium channels (VGCC) in primary cultures of hippocampal neurons from embryonic or newborn rats and mice (Kolarow et al. 2007; Kuczewski et al. 2008; Matsuda et al. 2009; Kojima et al. 2020). Thus, it could be speculated that BDNF release from intrinsically active neurons, which display uncorrelated calcium transients, or BDNF release from gap-junction-coupled neurons, which generate non-synaptic calcium-plateaus might play a role in shaping neuronal networks during embryonic and early postnatal development. However, this has not been extensively investigated so far.

To date, only GDPs were shown to represent robust activity patterns to induce release of BDNF in this context (Kuczewski et al. 2008; Mohajerani et al. 2007). Kuczewski et al. (2008) combined whole-cell recordings and time-lapse fluorescence imaging of transfected hippocampal neurons and demonstrated BDNF-GFP release after repeated depolarization steps that were strongly reminiscent of GDPs (Kuczewski et al. 2008). Furthermore, Mohajerani et al. reported plasticity of CA3-CA1 synapses after pairing of GDPs with Schaffer collateral stimulation in hippocampal slices during the first postnatal week of development (Mohajerani et al. 2007). They described that the depolarizing action of GABA during GDPs induced calcium influx through L-type VGCC, thereby triggering plasticity of CA3-CA1 synapses by endogenously released BDNF (Gubellini et al. 2005; Mohajerani et al. 2007). The depolarizing effects of GABAergic transmission fade out with ongoing postnatal development and this maturation of hippocampal networks requires a shift in the expression of chloride transporters in neurons from K^+^-Cl^−^-cotransporter (KCC2) to Na^+^-K^+^-chloride^−^-cotransporter (NKCC1), which switches GABA transmission to be hyperpolarizing. Since regulation of KCC2 expression is also mediated by BDNF (Rivera et al. 2002, 2005; reviewed in Fiumelli and Woodin 2007), depolarization-induced release of BDNF is important for proper shaping of hippocampal synaptic circuits.

#### Stimuli-triggering BDNF release from mature neurons

Depolarizing waves propagating through cortical networks represent on the one hand physiological activity patterns during neural circuit development but on the other hand, they have also been implicated in the pathophysiology of stroke, head trauma, or migraine aura (Hartings et al. 2017; Shen et al. 2016). Transient depolarizations of neurons induced by elevated extracellular K^+^-concentration have been described as a phenomenon called cortical spreading depression (CSD) or spreading depolarizations. CSD is characterized by waves of depolarizations that spread across the cortical surface at a low velocity of 2–5 mm/min. Consistent with activity-dependent regulation of BDNF, increases in BDNF mRNA and protein levels have been observed in response to CSD in rodents (Kawahara et al. 1997; Kokaia et al. 1993). Therefore, a protective role of CSD-induced BDNF expression and release enhancing the tolerance of further injury has been postulated (Kawahara et al. 1997; Shen et al. 2016). Interestingly, a very potent protocol to induce robust neuronal BDNF release is to elevate the extracellular K^+^-concentration, which in turn leads to depolarization and intracellular Ca^2+^-elevation. Such an elevated K^+^-induced release of BDNF was observed, e.g., in embryonic as well as postnatal neuronal cultures, neurons derived from embryonic stem cells and acute and organotypic hippocampal slices (Canossa et al. 1997; Goodman et al. 1996; Griesbeck et al. 1999; Hartmann et al. 2001; Kojima et al. 2001; Leschik et al. 2013; Leschik et al. 2019).

#### Stimuli-triggering BDNF release in synaptic plasticity

In addition to the development of brain circuits, BDNF is also involved in synaptic and network plasticity processes in mature circuits. Synaptic plasticity (i.e., LTP and LTD) is thought to be a cellular model of learning and memory processes. However, analyzing the activity patterns forming a memory together with the related structural changes of single synapses in a neuronal circuit in vivo is a challenging task. Although it is widely accepted that synaptic changes tune neural circuitry, the contribution of synaptic changes to memory encoding or memory consolidation is not resolved. Furthermore, the physiological correlates of the different activity patterns known to modulate synaptic efficacy, like high or low frequency stimulation, need to be identified. To date, several activity patterns shaping glutamatergic and GABAergic synapses either at pre- and/or postsynaptic sites have been investigated. The efficacy of the different patterns, of course, depends on diverse factors such as the developmental stage of the investigated neuronal circuit. The best-known patterns to induce long-term potentiation (LTP) of synaptic transmission in the hippocampus are high-frequency stimulation (HFS; e.g., repeated 1-s trains at 100 Hz), theta-burst stimulation (TBS; repeated bursts of 4–5 APs at ~ 100 Hz with inter-burst intervals of 0.2 s) or pairing protocols like spike timing-dependent plasticity (STDP; compare, e.g., Gottmann et al. 2009). Protocols that elicit long-term depression (LTD) of synaptic efficacy are characterized by low-frequency synaptic stimulation (LFS at 1 Hz). Both, LTP and LTD, are known to shape synapses at the functional, molecular and structural level. Activity-dependent release of endogenous BDNF or of fluorescently labeled BDNF has been intensively investigated as a cellular mechanism of LTP in brain slices and in neuronal cultures from different brain regions. Release of BDNF induced by HFS was shown, e.g., in PNS-cultured neurons (Balkowiec and Katz 2000), in hippocampal cultures (Balkowiec and Katz 2002; Hartmann et al. 2001; Kojima et al. 2001; Matsuda et al. 2009), amygdala (Meis et al. 2012) and cortico-striatal synapses (Jia et al. 2010), using either BDNF ELISA measurements or live cell imaging of fluorescently labeled BDNF. In contrast, similar patterns of high-frequency stimulation failed to induce efficient release of BDNF in dorsal horn slices from lumbal spinal cord (Lever et al. 2001). However, these authors were among the first to reveal TBS (300 bursts in 75 trains at 100 Hz with an interburst intervals of 0.2 s) as an effective means to induce release of BDNF (compare Balkowiec and Katz 2002). In addition to theta-burst discharges, BDNF release in spinal cord slices could also be elicited by injection of capsaicin, which in turn produces bursting activity reminiscent of theta bursts in the investigated nociceptors (Lever et al. 2001).

Bursting activity, which is repeated at a frequency of the theta rhythm, mimics also electrical activity patterns during hippocampal learning. Accordingly, TBS of Schaffer collateral CA1 synapses in acute brain slices was shown to efficiently release endogenous BDNF, thereby mediating postsynaptically expressed STDP (Edelmann et al. 2015). Correspondingly, TBS was also reported to release fluorescently labeled BDNF from either pre- or from postsynaptic sites in dissociated hippocampal cultures (Bergami et al. 2008; Matsuda et al. 2009) and from cortico-striatal presynaptic terminals (Park 2018). In addition to TBS-induced release of BDNF in hippocampal preparations, many other studies have analyzed the role of endogenously released BDNF in TBS-induced LTP, by either scavenging extracellular BDNF with superfusion of cells with selective BDNF antibodies, or by quantifying released BDNF by ELISA measurements in other brain regions. In this way, BDNF was shown to mediate TBS-LTP, e.g., at glutamatergic inputs to the amygdala (Li et al. 2011) or at retino-optical synapses (Du et al. 2009).

Although the BDNF-dependent LTP-inducing protocols seem to be very similar, the release sites of BDNF as well as the site of BDNF action depend on brain region and context (reviewed in Edelmann et al. 2014; Zagrebelsky et al. 2020). Moreover, even similar patterns of synaptic activity may induce mechanistically distinct types of LTP in the same neuronal circuit and subtle experimental details can determine in which way BDNF is involved in this plasticity. For example, spike timing-dependent long-term potentiation (t-LTP) is characterized by nearly coincident pairing of presynaptically induced excitatory postsynaptic potentials (EPSP) with postsynaptic firing of an action potential (AP) (Bi and Poo 1998). Such activity patterns were shown for the first time in the cortex of tadpoles to elicit BDNF release-dependent t-LTP (Mu and Poo 2006). However, slight changes in t-LTP protocols can decide whether t-LTP is mediated by BDNF or other modulators. In this respect, Edelmann et al. (2015) reported that a burst t-LTP protocol with 4 bAP was mediated by endogenously released BDNF, while t-LTP induced by stimulation protocols including only 1 bAP, occurred independent from BDNF release (Edelmann et al. 2015). These slightly different protocols did not occlude each other, indicating the differential physiological relevance of both protocols at the identical synaptic sites. In another study, regional deletion of BDNF either in the CA1 or the CA3 region revealed a role of presynaptically released BDNF in the induction of HFS-LTP, while postsynaptically released BDNF contributed to the maintenance of HFS-LTP (Lin et al. 2018). Complementary to the above-mentioned in vitro studies, Messaoudi et al. investigated the role of released BDNF in HFS-induced LTP in the hippocampus in vivo. Application of exogenous mature BDNF induced LTP (BDNF-LTP) in perforant path-granule cell synapses of the hippocampus, which occluded gene transcription dependent late-LTP but not early-LTP at the same synapses (Messaoudi et al. 2002). Future studies need to elucidate the role of proBDNF- and BDNF-dependent LTP in vivo in other synaptic circuits of the hippocampus and in additional brain areas (see also Zagrebelsky et al. 2020)

Subsequently to the release of BDNF from BDNF-expressing cells, the protein can also be released after endocytosis of the released BDNF (Fig. 5). Interestingly, the endocytosed BDNF can be further modified by the recipient cell before the re-release event. Such a re-exocytosis or recycling of BDNF has been reported for cultures of mature neurons, astrocytes and platelets (Bergami et al. 2008; Huang et al. 2014; Santi et al. 2006; Vignoli and Canossa 2017). In neurons, recycling of BDNF enables, on the one hand, the replenishment of the BDNF pool of releasing cells and re-use of BDNF after further processing steps. On the other hand, it also enables long distance distribution of synthetized BDNF across synaptically connected neuronal circuits and surrounding astrocytes after transcytosis of BDNF, a mechanism which was previously described or postulated for different neurotrophic factors (Bartheld and Johnson 2001; Butowt and Bartheld 2001; Wirth et al. 2005).

Endocytosis or re-uptake of BDNF is also an important mechanism to fill BDNF stores in platelets (Fujimura et al. 2002; Serra-Milàs 2016). These stores may constitute the major source of BDNF that can be released from platelets in response to blood vessel injury. Of note, the expression of BDNF not only in neurons but also in non-neuronal tissue particularly in the immune system and the cardiovascular system raises the interesting question how sorting and release of BDNF is regulated in these cells. Physiological stimuli, like shear stress in blood vessels but also pathophysiological and oncogenic stimuli, might orchestrate BDNF expression and release in these cell types as well as in cell types in which BDNF expression is under debate or even not proven so far. Future research addressing these important questions is critically needed.

### BDNF release from astrocytes

#### Stimuli-triggering BDNF from astrocytes during plasticity processes

BDNF can be secreted from neuronal cells but also from other cells such as astrocytes. Quantifying BDNF release by ELISA measurements during synaptic plasticity processes or analyzing the contribution of BDNF during LTP by scavenging BDNF from extracellular space (Shelton et al. 1995) do not clarify the original release sites of BDNF. Moreover, a potential permissive function of BDNF for LTP, or acute early or late instructive actions of BDNF during LTP, may depend on BDNF release from different cellular sources. In the central nervous system, astrocytes are closely associated with synapses. One main function of astrocytes is the modulation of synaptic function. It is known that proBDNF is endocytosed by astrocytes in a p75-dependent manner during TBS-LTP in hippocampal or perirhinal cortex slices (Bergami et al. 2008; Vignoli et al. 2016). Extracellular cleavage of proBDNF by plasmin or deletion of glial p75 receptors inhibits endocytosis of proBDNF by astrocytes (Bergami et al. 2008; Vignoli et al. 2016). After recycling of endocytosed proBDNF and intracellular cleavage, the re-released mature BDNF contributes to the maintenance of TBS-LTP in a postsynaptic TrkB-dependent manner (Vignoli et al. 2016). Furthermore, mice that were unable to recycle BDNF failed to recognize familiar from novel objects. Therefore, astrocytic BDNF recycling was shown to be essential for visual recognition memory (Vignoli et al. 2016). Besides the astrocytic recycling of BDNF, neuronal recycling of BDNF has also been shown to contribute to TBS-LTP maintenance in hippocampal slices (Santi et al. 2006). The role of astrocytic recycling has not been addressed in this study. Whether neuronal and astrocytic recyclings of BDNF coexist or whether they are successive processes need to be clarified. In contrast to the endocytosis of pro-BDNF, another study suggests that solely the mature form of BDNF is endocytosed by astrocytes via truncated TrkB receptors in dissociated cultures (Stahlberg et al. 2018). Interestingly, inflammatory stimuli like released interferon-gamma increased astrocytic expression of truncated TrkB, which was associated with increased BDNF recycling capacity of astrocytes (Rubio 1997). Additional studies are needed to clarify whether astrocytic recycling of mature BDNF via truncated TrkB-receptors contributes to astrocyte-dependent synaptic plasticity underlying memory formation or whether it is a part of inflammatory processes.

During plasticity processes, different stimuli might induce release of recycled BDNF from astrocytes. Since glutamate is known to induce the release of astrocytic BDNF (Jean et al. 2008; Santi et al. 2006; Vignoli et al. 2016), the prolonged glutamate release from the presynaptic terminal may be important for the release of recycled BDNF from astrocytes during TBS. These authors showed that the effect of glutamate was mimicked by agonists of AMPA or mGluRI/II receptors (Bergami et al. 2008). Interestingly, TBS activity is likely to induce elevated extracellular potassium levels. Consistent with this, BDNF release from astrocytes was also shown to be induced by elevated extracellular potassium concentration (Stahlberg et al. 2018; Vignoli and Canossa 2017). However, HFS was ineffective in releasing recycled BDNF from pure astrocytic cultures (Bergami et al. 2008).

Another stimulus to induce BDNF release was described by Canossa et al., in hippocampal slices and in neuronal cultures (Canossa et al. 1997). There, BDNF-induced BDNF release was observed (Canossa et al. 1997; Nakajima et al. 2008 but compare Kolarow et al. 2007). This mechanism might also occur in astrocytes during synaptic function to refill stores of BDNF and simultaneously to induce release of a readily releasable pool of BDNF vesicles. Taken together, processing and release of BDNF from astrocytes during synaptic plasticity process and memory formation are factors that should not be neglected.

#### Stimuli-triggering BDNF from astrocytes in inflammatory or neuroprotective processes

In addition to synaptic functions, astrocytes have several other functions in the CNS. They are important for the regulation of energy supply and for the homeostasis of neurotransmitters and ions. They are involved in function and maintenance of the blood brain barrier and they have immunoregulatory functions. Different stimuli are known to activate one or the other effect of astrocytes, respectively. One player that is associated with inflammation, pain, or immune response in astrocytes is prostaglandin E2 (PGE2). PGE2 has been shown to induce release of BDNF from astrocytes (Hutchinson et al. 2009). In addition to PGE2, the proinflammatory cytokine TNF-alpha was also shown to induce BDNF release from astrocytes (Giralt et al. 2010; Saha et al. 2006).

Furthermore, astrocytes are known to mediate neuroprotective functions, which involve BDNF release (Su et al. 2012). However, neuroprotective functions of astrocyte-derived BDNF were shown to be region-specific (Datta et al. 2018). These authors showed that cultured astrocytes from different brain regions exhibit distinct efficacy of BDNF release in response to oxidative stress induced by 6-hydroxydopamine (6-OHDA).

In line with the neuroprotective function, astrocytes are also important in regulating the glymphatic system. This waste clearance system of the brain has been described as a tunnel system around blood vessels formed by astrocytes. It is important for clearing the brain from toxic compounds like Amyloid-β-, which is itself a stimulus to induce BDNF release from astrocytes (Hou et al. 2011). However, neuronal release of BDNF was unaffected in transgenic Alzheimer models although transport of BDNF was impaired already after acute application of Amyloid-β (Seifert et al. 2016). In conclusion, regulation of the glymphatic system in response to Amyloid-β might be a BDNF-mediated neuroprotective effect of activated astrocytes in the brain.

### BDNF release from microglia

Similar to astrocytes, the primary immune cells of the brain, the microglia, also mediate several functions in the CNS, such as neuroprotection and shaping of synaptic spines (Dubbelaar et al. 2018; Stratoulias et al. 2019). Different microglia phenotypes exist, which can be transformed into one another by different chemokines and intercellular interactions, thereby exerting distinct functions in the brain (Orecchioni et al. 2019; Xue et al. 2014). One older simplifying in vitro classification of microglia, the M1/M2 concept, groups the different overlapping activated microglial phenotypes into two extremes: the proinflammatory lipopolysaccharide-(LPS)-induced M1 phenotype and the neuroprotective IL-4-induced M2 phenotype (Werry et al. 2019). BDNF expression and secretion from microglia have been shown for both activated M1 and M2 phenotypes but also for non-activated M0 microglia (Coull et al. 2005; Nakajima et al. 2001; Nakajima et al. 2002; Zhou et al. 2020). For the neuroprotective phenotype, extracellular accumulation of BDNF was shown after polarization of microglial cultures with IL-4 (Zhou et al. 2020). Furthermore, Merlo et al. demonstrated an Amyloid-β 42-induced release of BDNF thereby mediating a neuroprotective effect in cultures of a neuroblastoma cell line (Merlo et al. 2018).

LPS or 8-ceramide-induced polarization of microglia to the proinflammatory phenotype leads to an increase in extracellular mature BDNF that was described to depend on surface expression and activation of adenosine A_2a_ receptor (Gomes et al. 2013; Nakajima et al. 2002). Furthermore, surface expression of P2X4 adenosine receptors (P2X4R) drives microglia polarization towards the pro-inflammatory phenotype. The surface expression of P2X4Rs in microglia was associated with central sensitization like pain hypersensitivity or allodynia (Tsuda et al. 2013). Hypersensitivity in chronic pain was also associated with release of BDNF from microglia (Zhou et al. 2019). The uncovered molecular mechanism underlying BDNF-dependent hypersensitivity in chronic pain involved the small G protein MAPK p38 (Zhou et al. 2019). Activation of this protein is known to be important in P2X4R-mediated release of BDNF from microglia (Coull et al. 2005; Ferrini et al. 2013; Long et al. 2020; Trang et al. 2009). Activation of this purinergic ligand-gated cation channel (P2X4R) by extracellular ATP leads to an increase in intracellular calcium concentration, thereby triggering distinct molecular mechanisms to induce BDNF release from microglia.

## Molecular mechanisms underlying release of BDNF

### Calcium transients and BDNF secretion

**Fig. 1:**
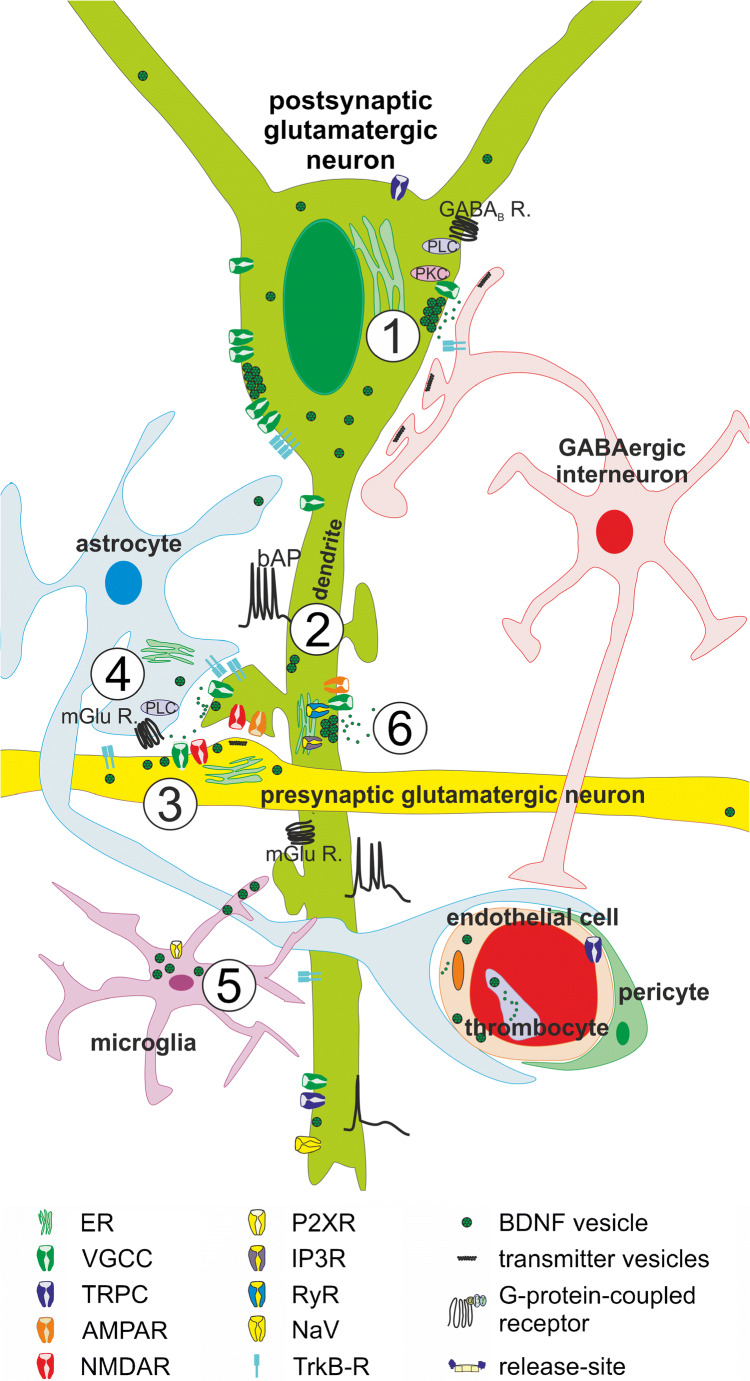
Schematic illustration of different release sites for BDNF. Release of BDNF takes place from somatic and dendritic compartments (green: ① + ②) and from axonal structures (yellow: ③) of glutamatergic neurons. Presynaptic neuron (yellow) and the postsynaptic glutamatergic neuron (green) are connected via glutamatergic synapses. The postsynaptic neuron additionally receives input from GABAergic interneurons (red). Astrocytic ④ and microglial ⑤ BDNF release has also been described. Recycling of BDNF ⑥ has been observed in neurons and in astrocytes. bAP back-propagating action potential, ER endoplasmic reticulum, GABA_B_R gamma-aminobutyric acid receptor B, IP3-R inositol trisphosphate receptor, mGluR metabotropic glutamate receptor, NaV voltage-gated sodium channel, NMDAR N-methyl d-aspartate receptor, P2XR P2X purinergic receptor, PKC protein Kinase C., PLC phospholipase C, TRPC transient receptor potential channel, VGCC voltage gated calcium channels. Adapted from Brigadski and Leßmann, Neuroforum, 2014.

Fusion of transmitter and peptide vesicles depends on calcium ions, which are essential for triggering membrane fusion and on ATP or GTP supply for energy consuming steps of exocytosis such as priming of vesicles and disassembly of the SNARE complex. In recent years, great efforts have been made to uncover the molecular mechanisms regulating exocytosis of BDNF-containing secretory granules. It is well established that the essential calcium transients to trigger membrane fusion may have extracellular and/or intracellular origin. Activation of NMDA receptors (Hartmann et al. 2001), AMPA receptors (Canossa et al. 2001; Hartmann et al. 2001), L-type, N-Type or P/Q-type voltage-gated calcium channels (Buldyrev et al. 2006; Kolarow et al. 2007), transient receptor potential channels (TRPC) (Vohra et al. 2013), or ATP-gated purinergic receptors (P2XR) (Long et al. 2020; Trang et al. 2009) have been shown to contribute to the calcium transients in different BDNF-releasing cell types (see Tables 1, 2,3 and Fig. 1). Moreover, calcium release from internal calcium stores crucially contributes to BDNF release (Kolarow et al. 2007) and is of particular importance for BDNF release in response to external stimuli acting on G protein-coupled receptors (GPCRs) or receptor tyrosine kinases (RTKs).

**Table 3:** Neuronal release—exact source of BDNF secretion is unknown, measurement of BDNF by, e.g., ELISA or scavenging of BDNF

Ref.	Species/type of preparation	Method	Release induction protocol	Pharmacology/molecular mechanism/time course
Wetmore et al. 1994	Rat	Immuno-histochemical staining	Kainat injection	Increase in DNQX dependent transcription of BDNF, irrespective of NMDA activation
Goodman et al. 1996	Rat; E16, hippocampus	Western blot	Potassium	Blocked in At20 cells in calcium-free solution
Figurov et al. 1996	Hippocampal slice	TrkB-IgG	HFS	
Kang et al. 1997	Hippocampal slice	TrkB-IgG	TBS	TrkB-IgG application 0 min and 30 min after LTP induction
			pairing	
Canossa et al. 1997	Rat; hippocampal culture E17 Hippocampal slice	ELISA	NT-4	Reduced by: K252; BAPTA-AM Not affected by: CNQX; BAPTA
			NT3	Reduced by: K252; BAPTA-AM Not affected by: CNQX; BAPTA
			Glutamate	Reduced to CTRL-level in the presence of CNQX One peak of BDNF release after 20 min Investigated time window: 100 min Not affected by K252a
Chen et al. 1999	Hippocampal slice	TrkB IgG	TBS	
Griesbeck et al. 1999	Hippocampal slice Hippocampal cultures from E17	ELISA	Potassium (up to 150%)	Blocked to ctrl-level in the presence of BAPTA-AM (intracellular calcium scavenging) Blocked to ctrl level in the presence of thapsigargin/caffeine Not effected in the presence of BAPTA + calcium free solution
			Glutamate	Blocked in the presence of BAPTA-AM (intracellular calcium scavenging) Not effected in the presence of BAPTA + calcium-free solution
Balkowiec and Katz 2000	Rat; cultures from nodose and petrosal ganglia neurons	ELISA	Potassium (72 h)	Accumulation of BDNF
			20 or 50 Hz stimulation	reduced by TTX No release after 5 Hz or 10 Hz stimulation
			50 Hz TBS	
Canossa et al. 2001	Hippocampal slice	ELISA	glutamate	No release after NMDA application
			AMPA	Reduced by: CNQX; caffeine + thapsigargin
			t-ACPD	Reduced by: AIDA; caffeine + thapsigargin
	Nnr5 cells Transfected with TrkA		NGF	Depedendent on exogenous TrkA expression Blocked by mutatet trkA with mutated PLC-site Reduced by BAPTA/AM; thapsigargin + caffeine
Patterson et al. 2001	Mouse; hippocampal slice	TrkB-IgG	TBS	Regulated by cAMP (reduction of forskolin-induced phosphorylation of TrkB in the presence of TrkB-IgG)
Balkowiec and Katz 2002	Rat; hippocampal neurons	ELISA	TBS	Blocked by TTX; conotoxin; extracellular Ca-free; dantrolene + thapsigargin; Not affected by: CNQX + APV; mGluR inhibitor; nimopidine
			t-ACPD (activator of mGluR)	Blocked by dantrolene + thapsigargin;
Canossa et al. 2002	Rat, hippocampal neurons; E17	ELISA	Network activity	Reduction up to 75% in the presence of glutamate receptor antagonists Increase up to 140% 20 min after L-NAME application Reduced after application of SNP (NO-donor); NOR3 (NO-donor); YC1 (agonist of sGC); 8 Br-cGMP Increased by KT5823 (inhibitor of PKG)
Gartner and Staiger 2002	Rat; hippocampal cultures from E19	ELISA	50 Hz train	Reduced by: TTX; Thapsigargin +caffeine; IP3R antagonist Not affected by: NMDAR antagonist; extracellular calcium-free solution
Egan et al. 2003	Rat, hippocampal culture E20 transfected with valBDNF or metBDNF	GFP-ELISA	Potassium	Reduced in neurons transfected with metBDNF-GFP
Aicardi et al. 2004	Hippocampal slice	ELISA	TBS-100 Hz	Release within 10 min after TBS Reduced BDNF release after LFS (5 Hz or 1 Hz)
Ba et al. 2005	Brain stem spinal cord preparations E16	ELISA	Basal level	Reduced by TTX; receptor antagonists Increased by elevated potassium
Buldyrev et al. 2006	Rat; Trigeminal neuronal culture	ELISA	Varying frequency	Reduced by: extracellular calcium-free solution; N-Type VGCC blocker; L-type blocker; P/Q-type VGCC blocker (reduction to 50% after inhibition of one of the receptor)
			CGRP	Reduced by: thapsigargin; thapsigarin + dantrolen Not affected by blocker of VGCC (N-, L-, P/Q-type)
Guo et al. 2006	Rat; brainstem/rostral ventromedial medulla slice	TrkB-phosphorylation; TrkB-IgG	TBS	
Santi et al. 2006	Hippocampal slice incubated with BDNF-YFP	Live cell imaging-release of endo-cytosed BDNF	Basal secretion	Not induced by NMDA application
				Not induced by NGF
				Reduced by NO donor NOR3
			Potassium	Reduced by k252a
			Glutamate	Recycling reduced by BAPTA-AM
			AMPA	Recycling reduced by CNQX
			t-ACPD	Recycling reduced by AIDA
			caffeine	
			NT-4	Reduced by K252a
			NT-3	Reduced by K252a
			50 Hz	Reduced by TTX
			KT5823 (inhibitor of PKG)	
Nakajima et al. 2008	Hippocampal E17 culture	FRET Cell-based fluorescent indicator for BDNF	BDNF 3.5 nm	Not affected by TTX; APV + CNQX
			Glutamate	Reduced by TTX; APV + CNQX
Tanaka et al. 2008	Organotypic hippocampal slice	TrkB-IgG	Uncaging glutamate + bAP	Not induced by unpaired stimulus
Babu et al. 2009	Mouse, hippocampal culture P0	ELISA	Glycine (60 min)	Glycine treatment do not induce NT-3 release
Du et al. 2009	Xenopus retinotectal system with pre and postsynaptic knockdown	Knockdown of site-specific TrkB expression	TBS	
Jourdi et al. 2009	Neuronal cultures	Western blot	CX641 (Ampakine)	Reduced by: CNQX, Ca-free solution; nifedipin; ryanodin
Fritsch et al. 2010	Motor cortex slice	TrkB IgG	Direct current stimulation (DCS)-LTP	
Hsieh et al. 2010	Nodose ganglion neuronal culture	ELISA	6-Hz stimulation	Reduced by NOR3 ➔ Rescued by NEM (prevention of s-Nitrosylation) not affected by: YC1 (guanylyl cyclase agonist); KT5823 (PKG antagonist); 8-Br-cGMP
				Reduced by SNAP (NO donor) ➔ rescued by Tempol (radical scavenger, prevention of S-nitrosylation)
Li et al. 2010	Hippocampal slice Mossy fiber ➔ CA3	TrkB IgG	TBS	MF(IAmpl) reduced in the presence of (glutamate and GABA receptor antagonists) + TTX
Li et al. 2011	Rat; brain slice	TrkB-Fc	TBS	LTP in BLA blocked by TrkB-Fc
Porcher et al. 2011	Cortical neurons	BDNF-IR	Muscimol (10 min)	Reduced by: bicuculine (back to control level)
			Ctrl	Reduced by: TTX: reduction to 50%
Meis et al. 2012	Coronal slices	TrkB-IgG	Pairing protocol: presynaptic 100 Hz + postsynaptic Depolarization	LTP blocked by APV LTP blocked by Pep1-TGL
Chen and Russo-Neustadt 2013	Rat; E18; hippocampal cultures	ELISA	Norepinephrine	Significant increase after 120 min
			5-HT	Significant increase after 10 min
			5 HT + norepinephrine	Significant increase after 5 min
Schildt et al. 2013	Adult mouse; hippocampal slice	TrkB-Fc	50 Hz stimulation	Reduced MF LTP in the presence of TrkB-Fc
Lepack et al. 2014	Rat; cortical culture; E18	ELISA	Ketamine	Reduced by verapamil (inhibitor of L-type VGCC) blocked by NBQ (inhibitor of (AMPAR)
Briz et al. 2015	Neuronal cultures	TrkB-Fc; Western blot	Estradiol (1 h)	
			G1 (1h)	G1 = G-protein-coupled estrogen receptor 1 agonist (GPER1) Not affected by ERalpha or ERbeta agonists PPT and DPN
Edelmann et al. 2015	Hippocampal slice	TrkB-IgG	Burst t-LTP	NMDAR-dependent
Zhao et al. 2015	Mouse; somatosensory cortex	TrkB blocking; BDNf rescue	TBS	
Hedrick et al. 2016	Rodent hippocampal slice	TrkB-Fc	Glutamate-uncaging	Heterosynaptic fascilitation of structural LTP
Atasoy et al. 2017	Rat E16	ELISA	Okadaic acid (24 h)	No change in BDNF release after Okadaic acid (8 h) Increase in BDNF level
Lao-Peregrin et al. 2017	Mouse, hippocampal slice	ELISA	Caffeine (5 min)	Reduced by TTX; ryanodin
Kato et al. 2018	Rat, primary cortical culture	Western blot; ELISA	GLYX-13 (allosteric modulator of NMDAR)	Reduced by verapamil
Lopez-Benito et al. 2018	DRG and cortical neurons, in vivo, in HD mice	ELISA	Basal levels	Increased by shRNA for ARMS
			Potassium	Increased by shRNA for ARMS
			NT-3	Increased by shRNA for ARMS
			NT-4	Increased by shRNA for ARMS
			Physical activity	In vivo BDNF release in different regions at different time points
Yu et al. 2018	Cortical neurons of zQ175 mice (HD model)	BDNF-pHluorin; live cell imaging	50 Hz	Smaller proportion of BDNF-containing vesicles undergoing full release
Zimbone et al. 2018	Rat; E15	ELISA	Basal release	Not affected by oligoAbeta
			Abeta1-42 (100 nM, 24 h)	Reduced by: selective inhibitor of IGF-IR (picropodophyllin) Not effected by: oligomeric Abeta1-42 Transcription dependent increase in BDNF level; increase in BDNF level to 150%
			IGF (5 ng/ml, 24 h)	Transcription dependent increase in BDNF level, increase in BDNF level to 190%
Fogaça et al. 2019	Primary cortical culture	ELISA	Methadone (60 min)	
Fukumoto et al. 2019	Cortical culture	ELISA	Hydroxynor-ketamine (60 min)	Reduced by verapamil (inhibitor of L-type VGCC); NBQX (inhibitor of (AMPAR)
Lee et al. 2019	Mouse, cortical neurons, Munc18 ko mice	ELISA	Basal level	Reduced in x/-munc18 ko culture
Liu et al. 2019	Rat	ELISA	Conditioned taste aversion memory extinction (12 h)	

List of references analyzing molecular mechanisms of BDNF release or the contribution of BDNF during LTP in different neuronal preparations. The exact cellular source of BDNF is unknown since BDNF was either quantified by, e.g., ELISA measurements or the significance of BDNF release was shown by scavenging BDNF from extracellular space. (Abbreviations: please see table legend of Table 1)

### Calcium transients and BDNF secretion in neurons

#### Calcium transients and BDNF secretion in response to electrical stimulation

**Fig. 2:**
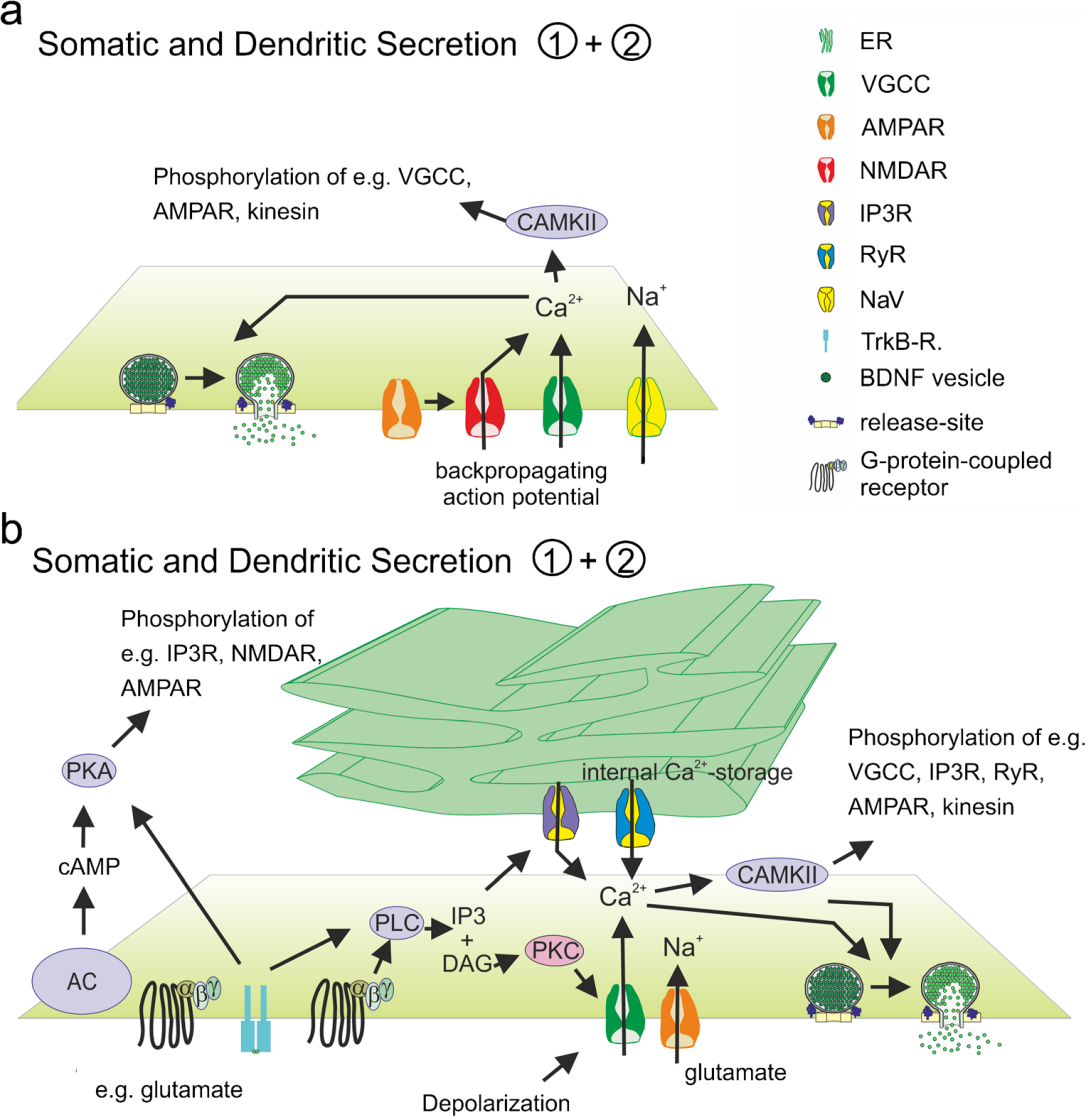
Suggested mechanisms for somatic and dendritic release of BDNF. BDNF release is dependent on extracellular Ca^2+^-influx (**a**) and/or intracellular Ca^2+^-release from internal stores (ER) Ca^2+^-influx (**b**). Ca^2+^-influx from extracellular space is mediated via VGCC and/or NMDAR (**a**, **b**). Ca^2+^ release from ER is mediated via IP3R or RyR (**b**). Increased burst firing activity, glutamate, or other ligands of GPCR mediate transient intracellular Ca^2+^-increase important for vesicle exocytosis. AC adenylate cyclase, CAMKII calmodulin-dependent protein kinase II; DAG diacylglycerol, ER endoplasmatic reticulum, IP3 inositol triphosphate, IP3R inositol-3-phosphate receptor, NaV voltage-gated sodium channel, NMDAR N-methyl d-aspartate receptor, PKA protein kinase A, PKC protein kinase C; PLC phospholipase C; RyR ryanodine, VGCC voltage-gated calcium channel. Adapted from Brigadski and Leßmann, Neuroforum, 2014

**Fig. 3:**
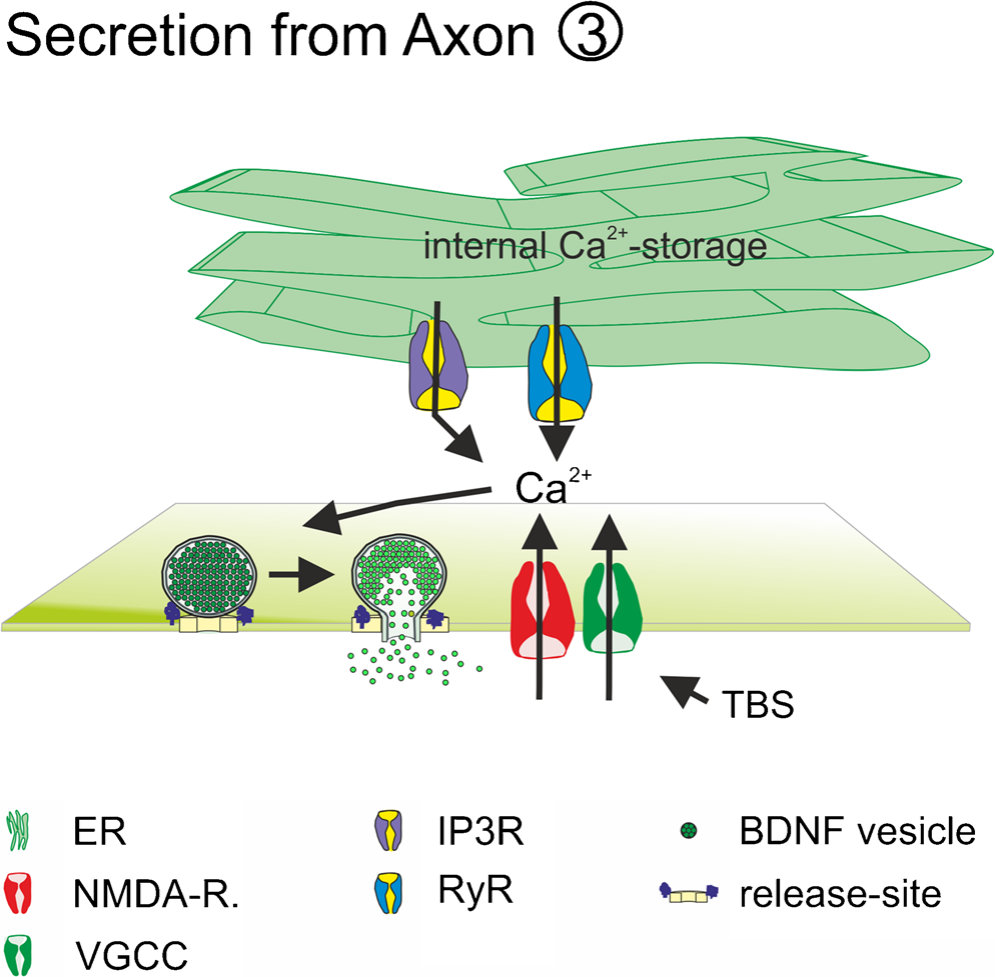
Suggested mechanisms for axonal release of BDNF. BDNF release is dependent on Ca^2+^-influx from extracellular space via presynaptic NMDAR and intracellular Ca^2+^-release from internal Ca^2+^-stores. ER endoplasmic reticulum, IP3R inositol-3-phosphate receptor, NMDAR N-methyl d-aspartate receptor, TBS theta burst stimulation

In neurons, calcium influx via pre- or postsynaptic NMDA receptors contributes to electrically induced BDNF release at the respective release site (Hartmann et al. 2001; Matsuda et al. 2009; Park 2018). At least for presynaptic terminals of cortico-striatal synapses, calcium influx from internal calcium stores was shown to prevent presynaptic BDNF release mediated via presynaptic NMDA receptors containing the GluN1 subunit (Park 2018) (Fig. 3). At postsynaptic release sites, electrically evoked BDNF release was solely dependent on extracellular calcium influx and not on calcium release from internal calcium stores (Kuczewski et al. 2008) (Fig. 2a). However, elevated potassium-induced BDNF release, depolarization-induced BDNF release and electrically evoked BDNF release in young cultures occur independent from AMPAR and NMDAR activation (Balkowiec and Katz 2002; Hartmann et al. 2001; Kuczewski et al. 2008) (Fig. 2b). In these cases, calcium influx was mediated via L-type VGCC (Balkowiec and Katz 2002; Buldyrev et al. 2006; Hartmann et al. 2001; Kolarow et al. 2007; Kuczewski et al. 2008), although the specific type of VGCC contributing to BDNF release might depend on the developmental stage and the brain area of the investigated neuron (Fig. 2b). Furthermore, dendritic elevated potassium-induced release of BDNF in hippocampal cultures was—in contrast to bAP-induced dendritic BDNF release—dependent on calcium efflux from internal calcium stores (Griesbeck et al. 1999; Kolarow et al. 2007; Kuczewski et al. 2008) (Fig. 2). This is in line with the role of BDNF during development of neural networks (compare Stimuli-triggering BDNF release from developing neurons section). The maturation of functional synapses might depend on BDNF release triggered by calcium-influx via VGCC and calcium release from internal calcium stores. In contrast, at more mature synapses, the calcium nanodomains defined by calcium influx via only NMDARs and VGCCs seem to be sufficient for bAP-induced site-specific local BDNF release from dendrites. Since calcium influx from internal stores was shown to be important for presynaptic AP-induced NMDAR-mediated BDNF release (Park 2018), calcium nanodomains might differ between dendrites and axons.

#### Calcium transients and BDNF secretion in response to chemical stimulation

Increased bursting activity of neural networks driven by glutamate or ketamine application or disinhibition of GABAergic transmission by GABA_A_R inhibitors represent potent stimuli for the induction of BDNF release (Canossa et al. 1997; Griesbeck et al. 1999; Lepack et al. 2014; Nakajima et al. 2008; Porcher et al. 2011; Santi et al. 2006). Interestingly, in contrast to bAP-induced BDNF release, glutamate-induced BDNF release from brain slices or primary neuronal cultures has been shown to be independent from extracellular calcium but dependent on calcium release from internal calcium stores (Canossa et al. 2001; Griesbeck et al. 1999; Santi et al. 2006) (Fig. 2b). This glutamate-induced BDNF release was also dependent on AMPA receptor or mGluR activation, respectively (Canossa et al. 1997; Canossa et al. 2001; Canossa et al. 2002; Nakajima et al. 2008; Santi et al. 2006), as well as on IP3-signaling that activates calcium release from internal calcium stores (Canossa et al. 2002; Gartner and Staiger 2002) (Fig. 2b). In most of these studies, BDNF levels in the extracellular medium was quantified by ELISA measurements. Therefore, release of BDNF from non-neuronal cells could not be excluded. Ketamine-induced BDNF release was blocked in the presence of AMPA receptor inhibitors but also in the presence of L-type VGCC inhibitors (Lepack et al. 2014). In this study, increased glutamatergic transmission induced by ketamine application was discussed as a reason for induction of BDNF release (Duman et al. 2019; Lepack et al. 2014). However, the molecular mechanisms underlying action of ketamine are not well understood (Lester et al. 2012; Wei et al. 2020). Therefore, it is possible that the ketamine-induced BDNF release acts via different mechanisms than glutamate-induced release. It is also conceivable that depolarization-induced conformational change of VGCC elicited by glutamate or ketamine-induced increase in bursting activity of the neural network is a prerequisite for BDNF release, which leads to structural reorganization of release, sites (Marom et al. 2010) irrespective of calcium influx from the extracellular medium.

#### Calcium transients and BDNF secretion in response to activation of GPCRs and RTKs

In addition to calcium influx through VGCC or NMDA receptors, calcium release from internal calcium stores is mediated by activation of G protein-coupled receptors (GPCRs) or receptor tyrosine kinases (RTKs). Different GPCRs and RTKs are known to be important for BDNF release. Neurotrophin-induced BDNF release was shown to depend on Trk receptor activation (Lopez-Benito et al. 2018; Nakajima et al. 2008; Santi et al. 2006). Furthermore, neurotrophin-induced BDNF release was solely dependent on calcium release from internal calcium stores (Canossa et al. 1997; Canossa et al. 2001; Lopez-Benito et al. 2018; Nakajima et al. 2008; Santi et al. 2006) (Fig. 2b). Similarly, GPCR-mediated BDNF release was also only dependent on calcium release from internal calcium stores (Buldyrev et al. 2006; Canossa et al. 2001; Lao-Peregrin et al. 2017; Santi et al. 2006). In addition, intracellular application of the non-hydrolysable guanosine diphosphate (GDP) analog GDPβ-S inhibits giant depolarization (GDP)-induced BDNF release (Kuczewski et al. 2008). In neuronal preparations, GCPRs activated by caffeine, glutamate via mGluR, GABA via GABA_B_ receptors, or calcitonin-gene-related protein (CGRP) receptor via CGRP have been shown to induce release of BDNF (Balkowiec and Katz 2002; Buldyrev et al. 2006; Canossa et al. 2001; Lao-Peregrin et al. 2017; Santi et al. 2006). Nevertheless, GPCR-mediated release seems to be even more prominent in non-neuronal cells.

#### Intracellular calcium transients and BDNF secretion from glial cells

**Fig. 4:**
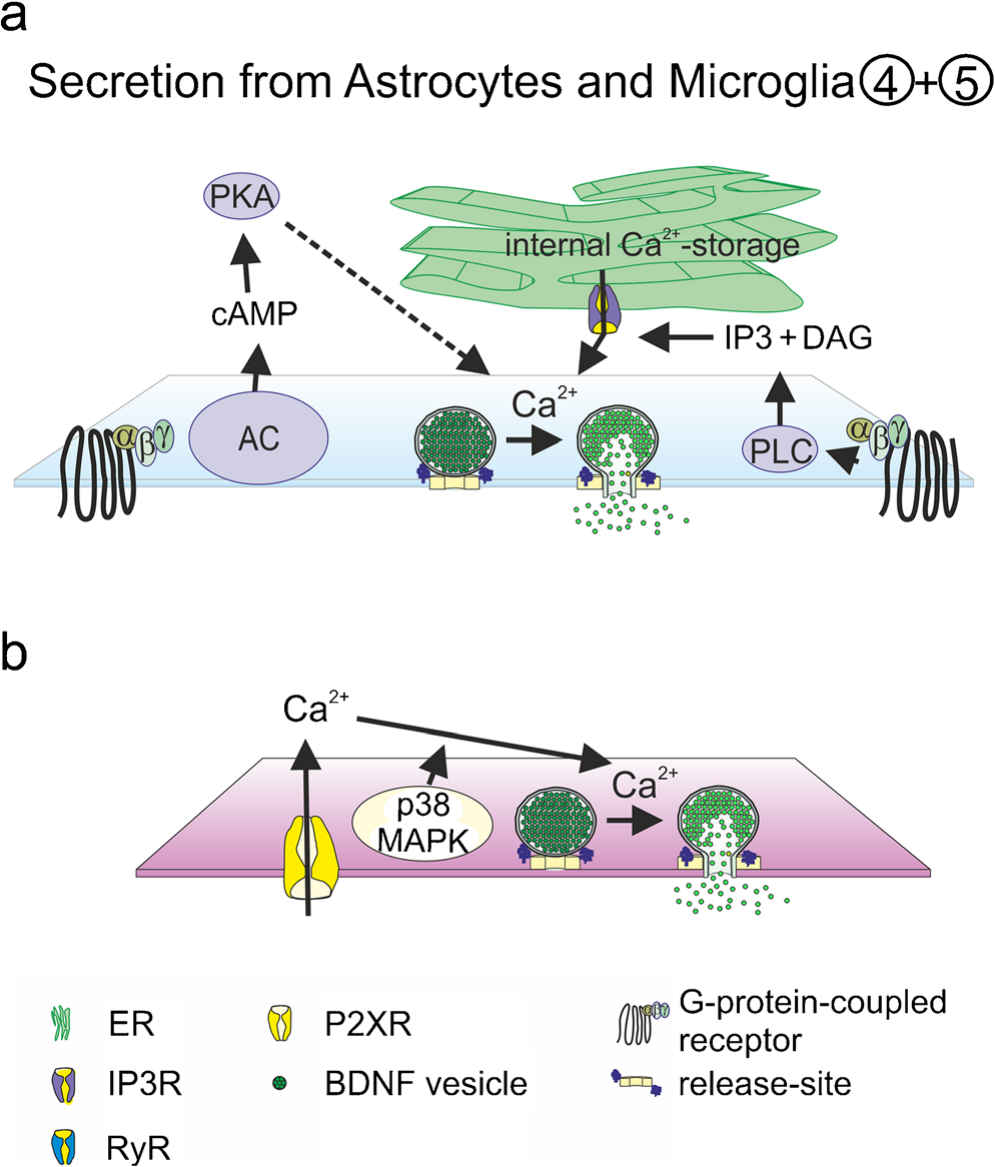
Suggested mechanisms for astrocytic and microglial release of BDNF. Glial BDNF release is dependent on GPCR activation and Ca^2+^-release from internal Ca^2+^-stores (**a**) and on Ca^2+^-influx via P2X-R (**b**). AC adenylate cyclase, DAG diacylglycerol, ER endoplasmatic reticulum, IP3 inositol triphosphate, IP3R inositol-3-phosphate receptor, p38MAPK p38-mitogen-activated protein kinase; P2XR purinergic P2X receptor, PKA protein kinase A, PLC phospholipase C; TRPC transient receptor potential channel

Ligands for GPCRs such as prostaglandine E2 or adenosine A2 (A_A2_R) induce BDNF release via activation of PKA or PLC in astrocytic and microglial cultures (Gomes et al. 2013; Hsieh et al. 2010; Hutchinson et al. 2009; Jean et al. 2008) (Fig. 4a). In addition to neuronal preparations, glutamate-induced release of BDNF was also observed in astrocytic (Jean et al. 2008) but not in microglial cultures (Trang et al. 2009). Glutamate-induced release of BDNF via mGluR-activation was observed within 10 min of stimulation, while kainate or NMDA application failed to induce release of BDNF in astrocytic cultures. The glutamate-induced release was dependent on activation of phospholipase C (PLC) and inositol-3-phosphate receptor (IP3-R) as well as on calcium release from internal calcium stores (Jean et al. 2008) (Fig. 4a). Besides the glutamate-induced BDNF release, release was also elicited after ATP treatment of astrocytic and microglial cultures (Long et al. 2020; Trang et al. 2009) (Fig. 4b). Trang et al. demonstrated a biphasic release of BDNF with peaks at 5 and 60 min after stimulation with ATP (Trang et al. 2009). SiRNA-mediated knockdown or pharmacological inhibition of purinergic P2X4Rs prevented the ATP-induced BDNF release (Long et al. 2020; Trang et al. 2009). Calcium influx from extracellular but not from internal calcium stores was important for BDNF release after ATP treatment (Fig. 4b). Application of TAT-NSF700, which prevents the disassembly activity of NSF, as well as inhibition of p38MAPK phosphorylation, reduced BDNF release from microglia (Long et al. 2020; Trang et al. 2009; Zhou et al. 2019). BDNF release from spinal dorsal horn microglia in response to p38-MAPK activation was associated with chronic pain hypersensitivity (Zhou et al. 2019). In addition to hydrophilic messengers, membrane permeable first messengers like progesterone or C8-ceramide are also effective stimuli to accumulate BDNF in extracellular medium after 16-h incubation time in primary glial cultures (Nakajima et al. 2002; Su et al. 2012).

### Contribution of small G proteins, SNARE proteins and signaling pathways to BDNF secretion

Different proteins are involved in the organization of the release site, the transition of BDNF-containing vesicles from the reserve pool to a readily releasable pool, fusion pore opening, as well as disassembly of the SNARE complex. The exact contribution of all the proteins and direct interactions between the proteins with all their multiple binding sites are unknown also for synaptic vesicles. For BDNF-containing granules, some proteins and their binding partners have been identified. Among them are important cytosolic kinases (Gimenez-Cassina et al. 2012; Gomes et al. 2013; Hsieh et al. 2010; Hutchinson et al. 2009; Kolarow et al. 2007; Santi et al. 2006; Trang et al. 2009), small G proteins (Hong et al. 2016; Kato et al. 2018; Persoon et al. 2019), SNARE proteins (Dean et al. 2009; Shimojo et al. 2015; Wong et al. 2015) and other priming factors (Eckenstaler et al. 2016; Lee et al. 2019).

#### Contribution of cytosolic-signaling pathways to BDNF secretion

Important cytosolic kinases, which are also implicated in BDNF release, are the protein kinase A (PKA), calcium calmodulin kinase II (CAMKII), protein kinase C (PKC) via phospholipase C (PLC), as well as protein kinase G (PKG) via the NO/cGMP/PKG-signaling pathway. The NO/cGMP/PKG-signaling pathway was shown to negatively regulate calcium release from internal calcium stores, thereby reducing BDNF secretion (Canossa et al. 2002; Kolarow et al. 2014; Santi et al. 2006). BDNF release was also dependent on activation of the PLC/IP3/IP3R-pathway, thereby mediating calcium release from internal calcium stores (Canossa et al. 2002; Gartner and Staiger 2002; Jean et al. 2008). Activation of PKA was shown to be important for BDNF release from neurons and glial cells (Gomes et al. 2013; Hsieh et al. 2010; Hutchinson et al. 2009; Kolarow et al. 2007) although the exact downstream effectors contributing to cAMP/PKA-dependent BDNF release are unknown. Furthermore, inhibition of CAMKII prevented BDNF release from neurons (Kolarow et al. 2007) while chronic inhibition of GSK-3 reduced accumulation of BDNF in culture media of neuroblastoma cells (Gimenez-Cassina et al. 2012). These serine/threonine kinases are known to regulate open channel probability of VGCCs, IP3Rs, the activity of effector proteins important for actin dynamics, as well as the activity of different other proteins, such as small G-proteins.

#### Contribution of small G-proteins to BDNF secretion

The Ras superfamily of small G proteins is an important regulator of several cellular processes. Based on structural and functional similarities, the superfamily is divided into 5 main subfamilies: Ras, Ran, Rho, Arf and Rab family. While the Ras family controls cell proliferation, differentiation and survival, the Ran family contributes to nucleocytoplasmic transport of RNA and proteins. The Rho family is important for actin dynamics and Rab and Arf subfamilies have been shown to regulate vesicular transport (Wennerberg et al. 2005). Different small G proteins seem to contribute to the release of BDNF. Activation of p38 MAPK is mediated via Ras. Ras/p38-signaling is important for BDNF release in astrocytes and microglia (Long et al. 2020; Trang et al. 2009). The small G-protein rab3a, which is important for vesicular trafficking, is localized on membranes of BDNF-containing granules, in astrocytes. Interactions between huntingtin (htt) and rab3a prevent binding of rab3a GTPase-activating protein1 (rab3a-GAP1) to rab3a, which leads to an impaired attachment of the granules to the plasma membrane (Hong et al. 2016). In synaptic vesicle release, it could be shown that Rab3a interacting protein Rim1alpha directly interacts with the priming factor munc13 thereby recruiting munc13 to the active zone. Inhibition of this interaction reduces the number of readily releasable vesicles (Andrews-Zwilling et al. 2006; Betz et al. 2001; Dulubova et al. 2005). A similar finding has been reported recently for BDNF-containing vesicles. Recruitment of BDNF-containing granules to release sites and thus fusion depends on the interaction of Rab3a-Rim1 and Munc13 (Persoon et al. 2019).

#### Contribution of regulatory proteins and SNARE proteins to BDNF secretion

The priming protein Munc13 further catalyzes the transition of the closed inhibitory form of the Munc18/syntaxin complex to the opened active form, thus enabling the formation of the SNARE complex. Therefore, Munc-18 is an essential protein for synaptic vesicle priming and stabilizing the core complex by binding to the SNARE protein syntaxin. Accordingly, in knockout mice heterozygous for munc-18, BDNF release is negatively affected (Lee et al. 2019).

The Ca^2+^-dependent activator protein for secretion (CAPS1) is another priming factor during exocytosis of synaptic vesicles and granule exocytosis in neurons (Farina et al. 2015; Jockusch et al. 2007). CAPS1, which is expressed in many brain regions, is localized in axons and dendrite of hippocampal neurons (Eckenstaler et al. 2016; Farina et al. 2015; Sadakata et al. 2006; Speidel et al. 2003). For BDNF-containing granules, a function of CAPS1 in regulating intravesicular pH value could be demonstrated (Eckenstaler et al. 2016). A transient knockdown of CAPS1 significantly increased the pH value in BDNF-containing granules while the pH in the cytosolic compartment was unaffected. Furthermore, knockdown of CAPS1 reduced the number of fusion-competent BDNF-containing vesicles as well as the amount of released BDNF per single vesicle by an unknown mechanism (Eckenstaler et al. 2016).

Furthermore, the contribution of different SNARE proteins to the release of BDNF was analyzed (Shimojo et al. 2015). In cortical cultures, the SNARE proteins involved in BDNF release slightly differ for axonal and dendritic release. While Syb2 and SNAP 25 were important for dendritic and axonal release of BDNF, the SNARE protein SNAP 47, which is irrelevant for synaptic vesicle exocytosis, contributed more to axonal than to dendritic BDNF release (Shimojo et al. 2015). Another protein that interacts with SNARE proteins is synaptotagmin (Syt). Synaptotagmins are localized in the vesicular membrane. One member of this protein family, synaptotagmin-4 (Syt-4), is upregulated by activity and seizure and also localized on BDNF vesicles. Syt-4 reduces depolarization-induced release of BDNF by preventing the fusion step (Dean et al. 2009). However, TBS-induced release of previously endocytosed BDNF was shown to be mediated by syt-6 and not syt-4 at postsynaptic sites in hippocampal neurons (Wong et al. 2015). Knockdown of the SNARE protein syt-6 as well as inhibition of direct interaction of syt-6 with the SNARE complex interacting protein complexin inhibited re-release of BDNF quantum dots. The recycling of BDNF was dependent on extracellular calcium as well as AMPAR or NMDAR activation, respectively (Wong et al. 2015). Wong et al. showed that endogenous and endocytosed BDNFs are both targeted to axons as well as to dendrites in distinct non-overlapping vesicles (Wong et al. 2015). While syt-6 is an important protein for recycling of BDNF in neurons, the release of endogenous post-Golgi BDNF was regulated by the SNARE protein syt-4 (Wong et al. 2015).

Altogether, great effort has been done to uncover the cellular and molecular mechanisms of BDNF release in recent years. A meshwork of proteins such as serine/threonine kinases, small G-proteins and SNARE complex (-interacting) proteins have been demonstrated to participate in regulated release of BDNF, while constitutive release of BDNF is mainly neglected in almost all studies. Since regulated release of synaptic vesicles and BDNF-containing vesicles share multiple proteins during fusion, the regulated release of vesicles seems to be a highly conserved evolutionary process.)

**Fig. 5:**
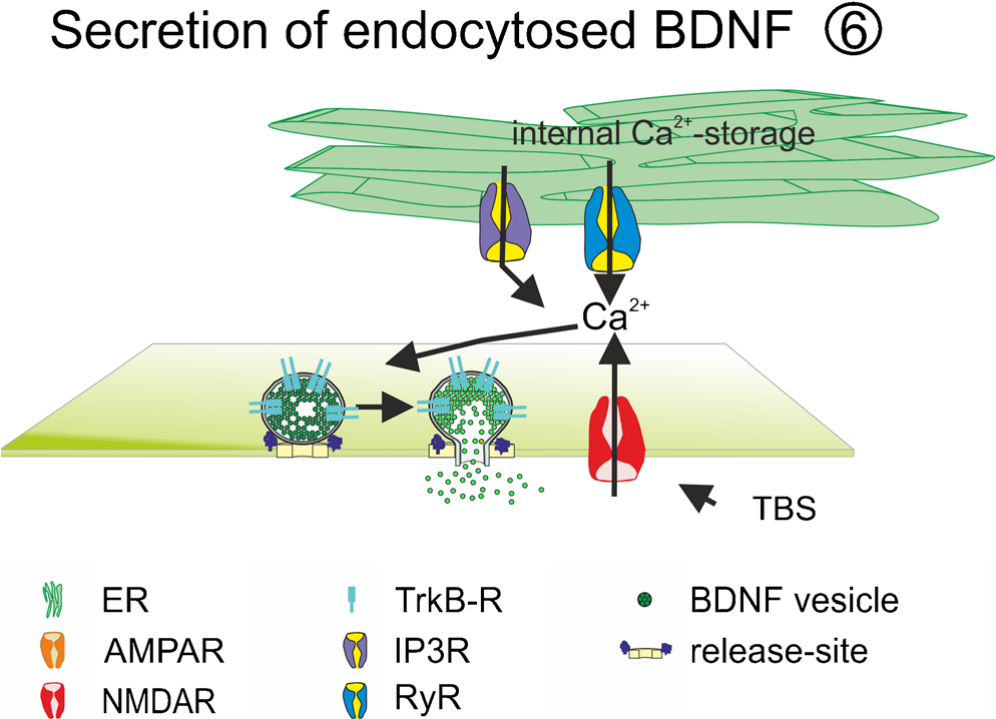
Suggested mechanisms for recycling of BDNF. Endocytosed BDNF is recycled for re-release event in neurons and astrocytes. ER endoplasmatic reticulum, IP3R inositol-3-phosphate receptor, NMDAR N-methyl d-aspartate receptor, TBS theta burst stimulation

## Conclusion

BDNF and the other neurotrophins were initially identified as target-derived survival factors that are secreted from non-neuronal tissue to secure survival of innervating neurons. In the early 1990s, the prominent function of BDNF in synaptic plasticity emerged, followed by first insights into activity-dependent release of BDNF from neurons. While sustained firing of action potentials or longer lasting depolarization are keys to neuronal BDNF secretion, the subsequent signaling cascades and the molecular machinery triggering and regulating BDNF vesicle exocytosis have just begun to be elucidated. Importantly, BDNF and potentially also the other neurotrophins can be released from many non-neuronal cell types using similar molecular mechanisms. Therefore, future studies will need to establish the following:

1. What are the physiological and pathophysiological correlates of the diverse BDNF-releasing stimuli in neuronal and non-neuronal cells?
2. How are the molecular pathways triggered in non-neuronal cells that can release BDNF (e.g.. microglia, astrocytes, lymphocytes, endothelial cells, muscle cells)?
3. What are the molecular differences between the release machinery for TGN-derived new vesicles vs. recycled vesicles from neuronal and non-neuronal cells, respectively?
4. What are the subcellular release sites of the distinct BDNF vesicles (new or recycled vesicles) in neuronal and non-neuronal cells?
5. What are the exact components of BDNF species (e.g., mBDNF, proBDNF and propeptide) and other cargo proteins (e.g., PCs, other neuropeptides, ATP and pH) in BDNF secretory granules that might help to fine tune the effects of released BDNF in target cells?
6. How do these differences in release sites, release machinery, distinct subsets of BDNF vesicles and components of BDNF species contribute to the distinct physiological and behavioral functions?
7. Last but not least: what is the origin and function of BDNF in the blood stream?
